# Conopeptides [V11L;V16D]ArIB and RgIA4: Powerful Tools for the Identification of Novel Nicotinic Acetylcholine Receptors in Monocytes

**DOI:** 10.3389/fphar.2018.01499

**Published:** 2019-01-07

**Authors:** Veronika Grau, Katrin Richter, Arik J. Hone, J. Michael McIntosh

**Affiliations:** ^1^Laboratory of Experimental Surgery, Department of General and Thoracic Surgery, German Centre for Lung Research (DZL), Giessen University, Giessen, Germany; ^2^Department of Biology, University of Utah, Salt Lake City, UT, United States; ^3^George E. Wahlen Department of Veterans Affairs Medical Center, Salt Lake City, UT, United States; ^4^Department of Psychiatry, University of Utah, Salt Lake City, UT, United States

**Keywords:** α-conotoxin, CHRNA7, CHRNA9, CHRNA10, immunomodulation, interleukin-1β, P2X7 receptor

## Abstract

Venomous marine snails of the genus *Conus* employ small peptides to capture prey, mainly osteichthyes, mollusks, and worms. A subset of these peptides known as α-conotoxins, are antagonists of nicotinic acetylcholine receptors (nAChRs). These disulfide-rich peptides provide a large number of evolutionarily refined templates that can be used to develop conopeptides that are highly selective for the various nAChR subtypes. Two such conopeptides, namely [V11L;V16D]ArIB and RgIA4, have been engineered to selectively target mammalian α7^∗^ and α9^∗^ nAChRs, respectively, and have been used to study the functional roles of these subtypes in immune cells. Unlike in neurons and cochlear hair cells, where α7^∗^ and α9^∗^ nAChRs, respectively, function as ligand-gated ion channels, in immune cells ligand-evoked ion currents have not been demonstrated. Instead, different metabotropic functions of α7^∗^ and α9^∗^ nAChRs have been described in monocytic cells including the inhibition of ATP-induced ion currents, inflammasome activation, and interleukin-1β (IL-1β) release. In addition to conventional nAChR agonists, diverse compounds containing a phosphocholine group inhibit monocytic IL-1β release and include dipalmitoyl phosphatidylcholine, palmitoyl lysophosphatidylcholine, glycerophosphocholine, phosphocholine, phosphocholine-decorated lipooligosaccharides from *Haemophilus influenzae*, synthetic phosphocholine-modified bovine serum albumin, and the phosphocholine-binding C-reactive protein. In monocytic cells, the effects of [V11L;V16D]ArIB and RgIA4 suggested that activation of nAChRs containing α9, α7, and/or α10 subunits inhibits ATP-induced IL-1β release. These results have been corroborated utilizing gene-deficient mice and small interfering RNA. Targeted re-engineering of native α-conotoxins has resulted in excellent tools for nAChR research as well as potential therapeutics. ^∗^indicates possible presence of additional subunits.

## Introduction

Nicotinic acetylcholine (ACh) receptors (nAChRs) are present at the neuromuscular junction in a wide variety of animal species. Venomous predators have evolved toxins targeted to neuromuscular nAChRs to facilitate prey capture and to defend against predators (Dutertre et al., 2014). Elapid snakes produce toxins characterized by a three finger protein domain (Fry et al., 2003; Utkin, 2013). These proteins are 60–80 amino acids in length, contain four disulfide bonds, and when injected produce paralysis in marine and terrestrial vertebrata. The vertebrate muscle nAChR subtype composed of α1, β1, δ, and ε/γ subunits has been intensively studied and extensive structure and function information has been obtained by examining the muscle nAChR in complex with three finger toxins, most notably α-bungarotoxin (Dellisanti et al., 2007).

Cone snails are among the dominant marine predators in coral reefs. Although their prey types include vertebrate fish, the predominant prey types of *Conus* species are invertebrates that include mollusks, polychaete, and hemichordate worms. Cone snails produce numerous types of conopeptides which are named, in part, according to their disulfide bond framework. Highly prevalent peptides include those that belong to the α-conotoxin family which target nAChRs (Abraham and Lewis, 2018; Giribaldi and Dutertre, 2018). Compared to elapid snake toxins, α-conotoxins are much smaller, usually 13–25 amino acids in length, and have only two disulfide bonds. Their small size facilitates *de novo* peptide synthesis of the native toxin as well synthesis of α-conotoxin-derived peptide analogs.

In addition to neurons, ACh is secreted and sensed by a broad range of non-neuronal cells including immune cells (Kawashima and Fujii, 2003, 2004; Wessler and Kirkpatrick, 2008; Beckmann and Lips, 2013; Kummer and Krasteva-Christ, 2014; Fujii et al., 2017a,b). The cholinergic system of immunity is a highly complex, regulated network that is capable of sending and receiving signals and can be modulated by other organ systems such as the central nervous system. We are only beginning to understand the cholinergic control of immunity that encompasses innate and adaptive immunity and can be pro- and anti-inflammatory (Fujii et al., 2017a,b). Essential components of the cholinergic system are expressed by immune cells in a regulated fashion, including transporters and enzymes involved in ACh synthesis, nAChRs, muscarinic ACh receptors, endogenous modulators of receptor function, and ACh-degrading esterases (Kawashima and Fujii, 2003, 2004; Fujii et al., 2017b). Immune cells express all five muscarinic ACh receptor subtypes (M1–M5) as well as nAChR subunits α2, α5, α6, α7, α9, α10, and β2 (Fujii et al., 2017b).

Similar to ACh, ATP can be released by nerve endings and function as a neurotransmitter (Burnstock, 2014). In addition, activated or damaged cells release cytoplasmic ATP into the extracellular space (Bortolotti et al., 2018). The most recognized and presumably most important function of extracellular ATP is that of a danger signal for monocytes/macrophages that leads to ion-channel functions of the ATP-receptor P2X7 (P2X7R) resulting in NLRP3 (NACHT, LRR, and PYD domains-containing protein 3) inflammasome assembly, activation of caspase-1, interleukin-1β (IL-1β) maturation, and release (Broz and Dixit, 2016; Bortolotti et al., 2018). IL-1β is a potent pro-inflammatory cytokine involved in host defense against infections (Broz and Dixit, 2016). However, IL-1β contributes to the pathogenesis of numerous debilitating diseases including autoimmune diseases and the life-threatening systemic inflammatory response syndrome (Dinarello et al., 2012; Bortolotti et al., 2018).

In this mini review, we summarize the strategies used to develop highly selective nAChR antagonists using native α-conotoxins as starting templates as well as their use in the discovery of an unexpected interaction of nAChR subunits α7, α9, and α10 in monocytic and epithelial cells. These unusual nAChRs efficiently control P2X7R activation, inflammasome assembly and, hence, release of IL-1β.

### Conopeptide Structure, Function and Development

There are ca. 700 species of *Conus*. Proteomic and transcriptomic analyses of *Conus* have demonstrated that there are likely thousands of unique α-conotoxins synthesized in the cone snail venom ducts (Lebbe et al., 2014; Robinson and Norton, 2014; Giribaldi and Dutertre, 2018). *Conus* thus represent an abundant source of lead compounds for conopeptide-based development. Peptide synthesis and pharmacological testing of α-conotoxins has shown that toxins from mollusk- and worm-hunting *Conus* lack potent activity at mammalian neuromuscular nAChRs and therefore are non-paralytic when injected into rodents. In contrast, some of these same α-conotoxins are potent antagonists of the nAChR subtypes expressed by neurons and non-neuronal cells (Azam and McIntosh, 2012) including those of immune cells as described in this review.

#### Development of the α7 nAChR-Selective [V11L;V16D]ArIB

*Conus arenatus* is a vermivore that hunts throughout the Indo-Pacific from East Africa to French Polynesia. Native ArIB was identified by genomic cloning from *C. arenatus* hepatopancreas and the predicted peptide synthesized (Whiteaker et al., 2007). Testing of ArIB on cloned nAChRs revealed potent activity on homomeric α7 (1.8 nM IC_50_) but also had substantial potency on α3β2 nAChRs (60 nM IC_50_). Structure-activity information from previously characterized α-conotoxins that have activity at α7 and α3β2 nAChRs was used to improve the selectivity of ArIB. Serial substitutions of the primary sequence of ArIB were made based on structure-activity studies of α-conotoxins PnIA and MII. PnIA inhibits both α7 and α3β2 nAChRs, but a single amino acid substitution of Leu for Val in position 10 of PnIA shifts activity in favor of α7 (Hogg et al., 1999; Luo et al., 1999). Substitution of Ala for Leu in position 15 of MII reduces activity for α3β2 nAChRs (McIntosh et al., 2004). We inserted both of these amino acids into the homologous position of ArIB to make [V11L;V16A]ArIB and determined that this analog had increased activity for α7 and decreased activity for α3β2 compared to native ArIB. Subsequent mutation of position 15 to Asp further lessened activity for α3β2. The final analog, [V11L;V16D]ArIB, had IC_50_ values of 1.1 nM for α7 and >10,000 nM IC_50_ for α3β2. [V11L;V16D]ArIB (Table 1) is the most selective α7 antagonist yet reported and is the basis for the generation of selective conopeptides with radioactive and fluorescent reporter groups (Whiteaker et al., 2008; Hone et al., 2009, 2010).

**Table 1:** Conopeptide Sequences.

Peptide	Sequence
ArIB^1^	DECCSNPACRVNNPHVCRRR
[V11L;V16D]ArIB^1^	DECCSNPACRLNNPHDCRRR
[A10L]PnIA^2^	GCCSLPPCALNNPDYC
[15A]MII^3^	GCCSNPVCHLEHSNAC
RgIA^4^	GCCSDPRCRYRCR
RgIA4^5^	GCCTDPRCX1X2QCY

*^1^Whiteaker et al., 2007; ^2^Luo et al., 1999; ^3^McIntosh et al., 2004; ^4^Ellison et al., 2006; ^5^Romero et al., 2017. X1, citrulline; X2, monoiodotyrosine; underlining indicates amino acid change from native peptide.*

#### Development of the α9α10 nAChR-Selective RgIA4

*Conus regius*, known as the crown cone, is found in the Caribbean Sea and in coastal waters of Brazil. *C. regius* preys on amphinomid worms and employs a small, 13 amino acid α-conotoxin known as RgIA. RgIA was shown to potently and selectively block rat α9α10 nAChRs (Ellison et al., 2006, 2008). In addition, RgIA was shown to treat and prevent the development neuropathic pain, suggesting a range of potential human therapeutic applications (Vincler et al., 2006; Di Cesare Mannelli et al., 2014; Pacini et al., 2016). Unfortunately, RgIA has low potency for human α9α10 nAChRs due to a Thr to Ile difference in the (-) binding face of the α9 subunit of the human α9α10 nAChR (Azam and McIntosh, 2012). In an attempt to overcome the low potency at the human nAChRs, non-Cys residues of RgIA were systematically substituted and the resulting analogs tested for activity. Residues in both the first and second disulfide loops of RgIA could be substituted to create analogs with increased potency for human α9α10 nAChRs. Four favorable substitutions were combined into one analog to create RgIA4 (Table 1). RgIA4 has low nM potency and high selectivity for human, mouse and rat α9α10 nAChRs (Christensen et al., 2017; Romero et al., 2017). Like RgIA, RgIA4 is effective at preventing and treating neuropathic pain in mice and rats (Christensen et al., 2017; Romero et al., 2017).

The α7 and α9 nAChR subunits have a close evolutionary relationship. Their similar sequences have made distinguishing among these subtypes difficult. α-Bungarotoxin and the plant norditerpenoid alkyloid methyllycaconitine both potently block α7 nAChRs but also have substantial potency for α9^∗^ nAChRs (Elgoyhen et al., 2001; Baker et al., 2004). With the advent of [V11L;V16D]ArIB and RgIA4, molecular dissection of α7 and α9^∗^ nAChR functions was enabled.

### Ion-Current Versus Metabotropic Functions of α7^∗^ NACHR and α9^∗^ NACHR

In most cases, nAChRs including α7^∗^ and α9^∗^ nAChRs function as ionotropic receptors that are permeable to the cations Na^+^, K^+^, and Ca^2+^ (Ullian et al., 1997; Katz et al., 2000; Verbitsky et al., 2000). α7^∗^ nAChRs are highly permeable to Ca^2+^ and display rapid desensitization characteristics which means a channel conformation state with high agonist affinity at the same time being impermeable to ions (Corradi and Bouzat, 2016).

Increasing evidence supports the existence of non-canonical signaling pathway(s) used by ligand-gated ion channels like nAChRs (Valbuena and Lerma, 2016). This metabotropic mode of action of nAChRs was first shown for the α7^∗^ nAChR in leukocytes. In T cells, activation of α7^∗^ nAChRs induced metabotropic signaling that resulted in an increase of intracellular Ca^2+^ concentrations independent of obvious ionotropic receptor functions (De Jonge and Ulloa, 2007; Razani-Boroujerdi et al., 2007). Similar channel-independent functions have also been shown in microglial cells (Suzuki et al., 2006; King et al., 2017) and in neurons (Zhong et al., 2008, 2013).

Proteomic analyses identified 55 intracellular interaction partners of α7^∗^ nAChR in the central nervous system and some of them may potentially mediate metabotropic signaling (Paulo et al., 2009). In addition, studies on neuronal cells indicate that α7^∗^ nAChRs are directly coupled to G-proteins and regulate axon growth at the growth cone (Kabbani et al., 2013; Kabbani and Nichols, 2018). G-protein mediated signaling in neuronal cells enables activation of growth-associated protein 43, as well as activation of phospholipase C, leading to inositol triphosphate-mediated release of Ca^2+^ from intracellular stores (Kabbani and Nichols, 2018).

In innate immune cells, various classical metabotropic signal transduction pathways and micro RNAs are involved in α7 nAChR-mediated down-regulation of pro-inflammatory cytokines and up-regulation of anti-inflammatory molecules at the transcriptional and translational level (Corradi and Bouzat, 2016; Fujii et al., 2017a; Hoover, 2017; Pavlov et al., 2018). In addition, one study suggests that extracellular ACh enters the cytoplasm, activates mitochondrial α7 nAChR and inhibits the release of mitochondrial DNA (Lu et al., 2014).

Whether stimulation of immune cells with nAChR agonists induces ion-channel functions is unclear. In most studies, no ion-currents have been detected in response to nAChR agonists (Peng et al., 2004; Razani-Boroujerdi et al., 2007; Hecker et al., 2009, 2015; Mikulski et al., 2010; Richter et al., 2016, 2018a; Zakrzewicz et al., 2017). However, stimulation of murine intestinal macrophages with agonists of α7 nAChR evoked small Ca^2+^ transients (Matteoli et al., 2014). It remains to be determined if these Ca^2+^ signals are due to ion-channel activity of nAChRs.

It has been suggested that metabotropic signal transduction through α7^∗^ nAChRs is associated with the desensitized conformation of the channel (Stokes et al., 2015; Corradi and Bouzat, 2016; Kabbani and Nichols, 2018). This suggestion is supported by the findings that some of the most effective modulators of α7^∗^ nAChR-mediated anti-inflammatory responses are compounds termed nAChR silent agonists, potent agonists of metabotropic functions in innate immune cells but do not evoke ionotropic functions (Thomsen and Mikkelsen, 2012; Chojnacka et al., 2013; Papke et al., 2014; Stokes et al., 2015; Horenstein and Papke, 2017).

### Cholinergic Control of Innate Immunity

#### Control of Gene Expression via α7^∗^ nAChR and α9^∗^ nAChR

A role of nAChRs in the regulation of innate immunity was first suggested by Tracey and colleagues, who reported that vagal nerve stimulation attenuates the release of the pro-inflammatory tumor necrosis factor in a model of endotoxin shock and coined the term “cholinergic anti-inflammatory pathway” (Borovikova et al., 2000). The anti-inflammatory effects were sensitive to an unspecified “α-conotoxin,” suggesting that signaling involves nAChRs (Borovikova et al., 2000). Subsequently, an essential role of α7 nAChR for the vagal control of inflammation was demonstrated (Wang et al., 2003). The current knowledge on cholinergic neuro-immune interactions involving α7 nAChR has recently been summarized in excellent reviews (e.g., Fujii et al., 2017a; Hoover, 2017; Pavlov et al., 2018). In addition to the nAChR α7 subunit, α9 and β2 subunits contribute to anti-inflammatory effects of nAChR agonists (Simard et al., 2013; Jiang et al., 2016; St-Pierre et al., 2016; Liu et al., 2017). It is, however, unclear if these nAChR subunits interact or if they independently trigger anti-inflammatory mechanisms.

#### Inhibition of P2X7R Ion-Channel Function by Conventional nAChR Agonists

**FIGURE 1:**
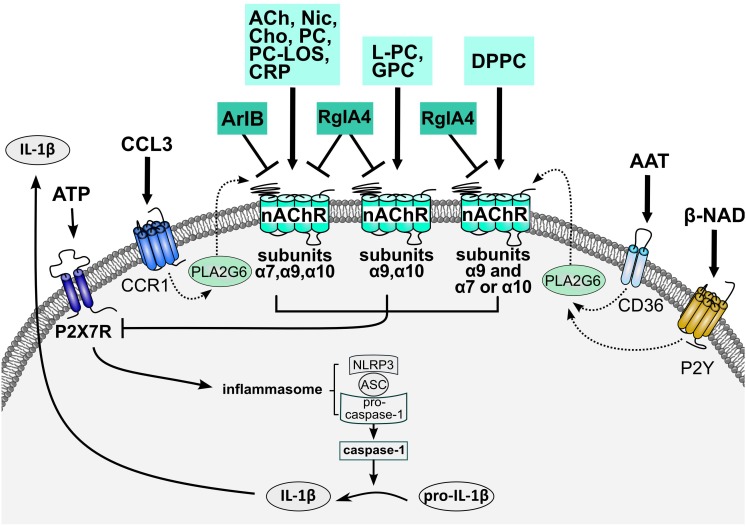
Working of the cholinergic control of ATP-dependent release of monocytic IL-1β. Stimulation of the ATP-gated P2X7R results in the assembly of the NLRP3 inflammasome and activation of capspase-1 that cleaves pro-IL-β and enables its swift release. Agonists of monocytic nAChRs metabotropically inhibit the ionotropic function of P2X7R and, hence, eventually IL-1β release. Different nAChR subunits interact, depending on the respective nicotinic agonist. Conventional nAChR agonists (ACh, Cho, and Nic) as well as PC, PC/CRP complexes and PC-LOS require nAChR subunits α7, α9, and α10 for signaling. LPC and G-PC depend on the interaction of nAChR subunits α9 and α10, whereas only nAChR subunit α9 is essential for signaling of DPPC. In the latter case, nAChR subunit α9 interacts with either subunit α7 or α10. Accordingly, signaling of ACh, Cho, Nic, PC, PC/CRP complexes, and PC-LOS is sensitive to both conopeptides, [V11L;V16D]ArIB and RgIA4, whereas signaling of L-PC, GPC and DPPC is only sensitive to RgIA4. These cholinergic control mechanisms are also triggered by the chemokine CCL3 that signals via chemokine receptor CCR1, activates PLA2G6, and induces the release of a yet unknown agonist of nAChRs composed of subunits α7, α9, and α10. In a similar way, AAT and β-NAD signal via CD36 and P2Y receptors and trigger the secretion of a nAChR agonist that activates nAChRs similar to DPPC. The structure of the nAChRs involved in the control of IL-1β release remains to be elucidated as well as the signaling cascade resulting in P2X7R inhibition. AAT, α1-antitrypsin; ACh, acetylcholine; ASC, apoptosis-associated speck like protein containing a caspase recruitment domain; Cho, choline; CRP, C-reactive protein; DPPC, dipalmitoyl phosphatidylcholine; GPC, nAChR, nicotinic acetylcholine receptor; Nic, nicotine; NLRP3, NACHT, LRR and PYD domains-containing protein 3; P2X7R, ATP receptor P2X7; PC, phosphocholine; PC-LOS, PC-modified lipooligosaccharides; PLA2G6, calcium-independent phospholipase A2β.

ATP-dependent IL-1β release by lipopolysaccharide-primed human monocytic U937 cells, primary human blood monocytes as well as human and mouse peripheral blood mononuclear cells (PBMCs) is efficiently inhibited by the nAChR agonists nicotine and ACh, but also by choline, a selective agonist of α7^∗^ and α9^∗^ nAChRs (Hecker et al., 2015; Richter et al., 2016; Figure 1). In line with an involvement of α7^∗^ and α9^∗^ nAChRs, inhibition of IL-1β release is sensitive to mecamylamine, α-bungarotoxin and strychnine (Hecker et al., 2015; Richter et al., 2016; Zakrzewicz et al., 2017). As these nAChR antagonists do not differentiate between α7^∗^ or α9^∗^ nAChRs, the conopeptides [V11L;V16D]ArIB and RgIA4 have turned out to be invaluable tools. Surprisingly, both conopeptides reversed the inhibitory effects of nicotine and ACh (Hecker et al., 2015; Zakrzewicz et al., 2017), suggesting an involvement of nAChR subunits α7, α9, and/or α10. Gene knock-down in U937 cells and knock-out mice revealed an obligate role of nAChR subunits α7, α9, and α10 in signaling (Hecker et al., 2015; Zakrzewicz et al., 2017).

Of note, nAChR agonists do not provoke obvious ion-channel functions in U937 cells as measured by whole-cell patch-clamp recordings, but completely abolish the ion-currents induced by P2X7R activation (Hecker et al., 2015; Richter et al., 2016). This is of eminent clinical importance, because nAChR agonists control sterile, trauma-associated inflammation without completely inhibiting host defense against pathogens that stimulate numerous ATP-independent pathways of IL-1β maturation (Broz and Dixit, 2016). The mechanism down-stream of nAChR activation controlling P2X7R ion-channel function is currently under investigation.

#### Phosphocholine Is an Agonist of Monocytic nAChR

Apart from conventional nAChR agonists, phosphocholine stimulates monocytic nAChRs and inhibits ATP-induced IL-1β release (Hecker et al., 2015; Richter et al., 2016, 2018a,b; Figure 1). The response of monocytic cells to free phosphocholine resembles that of choline: IC_50_ values are in the range of 10 μM, signaling involves nAChR subunits α7, α9, and α10, both compounds do not elicit ion-currents at U937 cells, but inhibit the ion-channel function of the P2X7R (Hecker et al., 2015; Richter et al., 2016). In sharp contrast to choline, phosphocholine does not induce ion-current responses in *Xenopus laevis* oocytes that heterologously express human nAChR α9 subunits, alone or co-injected with α7 and/or α10 (Richter et al., 2016; Zakrzewicz et al., 2017). Remarkably, choline-gated currents in *Xenopus* oocytes expressing human α9α10 nAChR are strongly but reversibly inhibited by phosphocholine, resembling silent agonist or antagonist functions (Richter et al., 2016). Hence, metabotropic functions of monocytic nAChRs can be elicited by endogenous agonists that do not induce ion-currents at conventional receptors. Whether phosphocholine functions as silent agonist of canonical α9^∗^ nAChR *in vivo*, remains to be investigated.

#### C-Reactive Protein (CRP) Potentiates the nAChR Agonist Function of Phosphocholine

The pentameric acute-phase protein CRP is mainly synthesized in the liver in response to increased circulating levels of IL-1β and IL-6. Under physiological conditions, CRP forms Ca^2+^-dependent complexes with phosphocholine and other compounds with a phosphocholine head-group at a stoichiometric proportion of 1:1 per monomer (Pepys and Hirschfield, 2003; Mantovani et al., 2008). Native CRP-ligand complexes are potent nAChR agonists at human monocytic cells that inhibit the ATP-dependent inflammasome assembly (Figure 1) and IL-1β release, whereas CRP devoid of ligands is ineffective (Richter et al., 2018a). The IC_50_ of CRP isolated from human bodily fluids is about 40 nM, far below that of phosphocholine (10 μM), suggesting that CRP potentiates the effect of free phosphocholine (Richter et al., 2018a). The effects of CRP-phosphocholine complexes on monocytic cells are sensitive to [V11L;V16D]ArIB and RgIA4, depend on interaction of nAChR subunits α7, α9, α10, and resemble silent agonists or partial antagonists at canonical α9α10 nAChR (Richter et al., 2018a). A prospective clinical study on patients suffering from multiple traumata was in line with a protective anti-inflammatory function of CRP *in vivo*, suggesting that endogenous CRP is a negative feed-back regulator of IL-1β-mediated inflammation (Richter et al., 2018a).

#### Phosphocholine-Modified Macromolecules Function as nAChR Agonists

Some eukaryotic parasites and bacterial pathogens conjugate phosphocholine moieties to proteins or cell wall glycolipids (Grabitzki and Lochnit, 2009; Clark and Weiser, 2013). Two opposing but not necessarily mutually exclusive views on the biological relevance of these PC-modified molecules prevail. First, CRP and highly prevalent phosphocholine-specific antibodies bind to phosphocholine-modified surfaces and activate mechanisms of pathogen elimination (Scott et al., 1987; Nishinarita et al., 1990; Shaw et al., 2000; Pepys and Hirschfield, 2003; Mantovani et al., 2008; De Faire and Frostegård, 2009; Frostegård, 2010; Fiskesund et al., 2014). Second, PC-modified products exert strong anti-inflammatory effects and serve the immune evasion of pathogens (Grabitzki and Lochnit, 2009; Clark and Weiser, 2013). We recently demonstrated that phosphocholine-modified lipooligosaccharides from wildtype *Haemophilus influenzae* are potent nAChR agonists inhibiting the ATP-induced release of IL-1β in monocytic U937 cells (25 nM IC_50_), in pulmonary epithelial cell lines and in living lung slices (Hecker et al., 2015; Richter et al., 2018b; Figure 1). Similarly, phosphocholine synthetically conjugated to bovine serum albumin, a compound that mimics the properties of secreted phosphocholine-modified helminth proteins, is a functional agonist of monocytic nAChR (Hecker et al., 2015).

#### Phosphatidylcholines and Their Metabolites Are Agonists at Monocytic α9^∗^ nAChRs

Phosphatidylcholines, common constituents of biomembranes and surfactant, are amphiphilic neutral lipids composed of a phosphocholine head-group linked to glycerol with two variable fatty acid chains. Dipalmitoyl phosphatidylcholine, the dominating lipid component of pulmonary surfactant (Lang et al., 2005; Lopez-Rodriguez and Pérez-Gil, 2014), inhibits ATP-induced IL-1β release (10 μM IC_50_) (Backhaus et al., 2017; Figure 1). This function is sensitive to RgIA4 but remarkably not to [V11L;V16D]ArIB (Backhaus et al., 2017). Accordingly, knock-down of nAChR subunit α9 blunts the effect of dipalmitoyl phosphatidylcholine, whereas single knock-down of nACh subunits α7 and α10 does not. However, concomitant knock-down of nAChR subunits α7 and α10 abolishes the effect of dipalmitoyl phosphatidylcholine suggesting that nAChR subunit α9 is mandatory for signaling, whereas α7 and α10 can replace each other (Backhaus et al., 2017).

Phosphatidylcholine metabolites palmitoyl lysophosphatidyl-choline and glycerophosphocholine, are also efficient inhibitors of ATP-mediated IL-1β release (1 μM IC_50_), their effects are sensitive to RgIA4 but not to [V11L;V16D]ArIB and they function as silent agonists or partial antagonists at canonical α9α10 nAChR (Zakrzewicz et al., 2017; Figure 1). In contrast to dipalmitoyl phosphatidylcholine, however, nAChR subunits α9 and α10 are mandatory for signaling of palmitoyl lysophosphatidylcholine and glycerophosphocholine, whereas α7 is dispensable (Zakrzewicz et al., 2017). It remains to be investigated, if these findings apply to all phosphatidylcholines and lysophosphatidylcholines or if the structure of the fatty acid remnants is of functional importance.

#### Signaling of Chemokines, β-NAD and α1-Antitrypsin Induce the Secretion of Agonists of Monocytic nAChR

Chemokines, β-NAD and α1-antitrypsin surprisingly inhibit the ATP-induced release of IL-1β via mechanisms that join the above described cholinergic pathways (Amati et al., 2017; Hiller et al., 2018; Siebers et al., 2018; Figure 1). CCL3 signals via chemokine receptor CCR1, activates the Ca^2+^-independent phospholipase A2β (PLA2G6) and induces the release of low molecular mass factors that function as agonists of monocytic nAChR. Signaling is sensitive to [V11L;V16D]ArIB and RgIA4 suggesting that subunits α7, α9, and/or α10 are required (Amati et al., 2017). β-NAD activates purinergic receptors P2Y1 and P2Y11, whereas α1-antitrypsin signals via CD36 to activate PLA2G6 and to induce the release of nAChR agonists (Hiller et al., 2018; Siebers et al., 2018). Surprisingly, these factors are different from those secreted in response to CCL3, as they are sensitive to RgIA4 but insensitive to [V11L;V16D]ArIB. Results from gene silencing experiments are in line with the assumption that β-NAD and α1-antitrypsin signal via nAChR subunits α9 and either α7 or α10 (Hiller et al., 2018; Siebers et al., 2018). The structure of these secreted factors remains to be determined. As the preferred substrates of PLA2G6 are phosphatidylcholines (Ramanadham et al., 2015), their above described metabolites are possible candidates. This novel triple-membrane passing signaling pathway involving PLA2G6 activation and nAChRs might be of general importance, far beyond the control of inflammasome activation.

#### Do Monocytic nAChRs Structurally Differ From Canonical nAChRs?

It is an open question as to how monocytic nAChRs inhibit ion-channel function of P2X7Rs. There are, however, several hints that monocytic nAChRs differ from classical pentameric channels. As they induce metabotropic functions (Hecker et al., 2009, 2015; Mikulski et al., 2010; Richter et al., 2016), there may be no need for the activatable state of a classical pentameric ion channel. In this instance, binding of agonist to the nAChR promotes a receptor state that allows G-protein coupling but not fast ionotropic conduction. Whereas nicotine acts as an agonist that induces ion-channel function at α7 nAChRs, for α9^∗^ nAChRS expressed in cochlear hair cells and those heterologously expressed in *Xenopus* oocytes, nicotine acts as an antagonist (Lustig et al., 2001; Sgard et al., 2002). In contrast, nicotine functions as an agonist at monocytic α9^∗^nAChRs. This might be due to the addition of an α7 subunit to the α9-containing receptor complex. Alternatively, pentameric nAChRs can exist in a state that is ‘uncoupled’ from ion-conducting function, yet might maintain metabotropic properties (Drenan et al., 2008; Baenziger et al., 2015). It is also possible that α9 subunits form non-pentameric oligomers together with subunits α7 and/or α10 that lack ionotropic function but possess metabotropic function.

Structural modeling revealed an accumulation of charged amino acids at the α9 (-) side that seems to interfere with loop-C closure and might hinder nicotine engulfment by the ligand binding pocket (Giastas et al., 2018). Because nicotine and bulky molecules such as palmitoyl lysophosphatidylcholine, dipalmitoyl phosphatidylcholine, CRP-phosphocholine complexes, phosphocholine-modified lipooligosaccharides, and phosphocholine-modified bovine serum albumin function as agonists (Hecker et al., 2015; Backhaus et al., 2017; Zakrzewicz et al., 2017), we speculate that binding sites of monocytic nAChR do not necessarily close upon ligand binding. May be, they even do not involve the (-) side of an adjacent subunit.

## Conclusion

*Conus* produce a vast array of toxins some of which have been systematically modified to produce peptides highly selective for mammalian nAChR subtypes. These conopeptides have been used to help demonstrate that monocytes express novel unexpected nAChRs that contain α9, α7, and/or α10 subunits, inhibit the ionotropic function of P2X7R and modulate ATP-induced IL-1β release. A diverse set of key compounds, some of them already known to modulate immune responses, act as agonists of these nAChRs suggesting a pivotal role in health and disease processes.

## Author Contributions

VG, KR, AH, and JMM wrote the article. VG and KR designed the figure.

## Conflict of Interest Statement

Certain conopeptides, including RgIA4 have been patented by the University of Utah; JMM is an inventor on these patents. The remaining authors declare that the research was conducted in the absence of any commercial or financial relationships that could be construed as a potential conflict of interest.
